# Store-Operated Ca^2+^ Entry as a Prostate Cancer Biomarker — a Riddle with Perspectives

**DOI:** 10.1007/s40610-017-0072-8

**Published:** 2017-10-28

**Authors:** Sven Kappel, Ines Joao Marques, Eugenio Zoni, Paulina Stokłosa, Christine Peinelt, Nadia Mercader, Marianna Kruithof-de Julio, Anna Borgström

**Affiliations:** 1 Institute of Biochemistry and Molecular Medicine, NCCR TransCure, University of Bern, Bern, Switzerland; 2 Institute of Anatomy, University of Bern, Bern, Switzerland; 3 Urology Research Laboratory, Department of Urology and Department of Clinical Research, University of Bern, Bern, Switzerland; 4 Department of Urology, Leiden University Medical Centre, Leiden, The Netherlands

**Keywords:** Ion channel, Prostate cancer, Store-operated calcium entry, Prostate cancer stem cells, Zebrafish

## Abstract

**Purpose of Review:**

Store-operated calcium entry (SOCE) is dysregulated in prostate cancer, contributing to increased cellular migration and proliferation and preventing cancer cell apoptosis. We here summarize findings on gene expression levels and functions of SOCE components, stromal interaction molecules (STIM1 and STIM2), and members of the Orai protein family (Orai1, 2, and 3) in prostate cancer. Moreover, we introduce new research models that promise to provide insights into whether dysregulated SOCE signaling has clinically relevant implications in terms of increasing the migration and invasion of prostate cancer cells.

**Recent Findings:**

Recent reports on Orai1 and Orai3 expression levels and function were in part controversial probably due to the heterogeneous nature of prostate cancer. Lately, in prostate cancer cells, transient receptor melastatin 4 channel was shown to alter SOCE and play a role in migration and proliferation. We specifically highlight new cancer research models: a subpopulation of cells that show tumor initiation and metastatic potential in mice and zebrafish models.

**Summary:**

This review focuses on SOCE component dysregulation in prostate cancer and analyzes several preclinical, cellular, and animal cancer research models.

## Introduction

Physiological calcium concentrations vary widely in different intra- and extracellular compartments. While the extracellular Ca^2+^ concentration is about 1.2 mM, the cytoplasmic Ca^2+^ concentration ranges from the 100 nM to μM range. In the endoplasmic reticulum (ER), which functions as an intracellular Ca^2+^ store, the Ca^2+^ concentration is up to ~ 500 μM. Changes in intracellular Ca^2+^ act as signals that drive various cellular functions, including gene expression and cell migration, proliferation, and apoptosis [1–6]. In addition, numerous Ca^2+^ transporting enzymes generate tightly regulated local Ca^2+^ subdomains that are important for enzymatic and cellular functions, especially cell migration [7, 8].

**Fig. 1:**
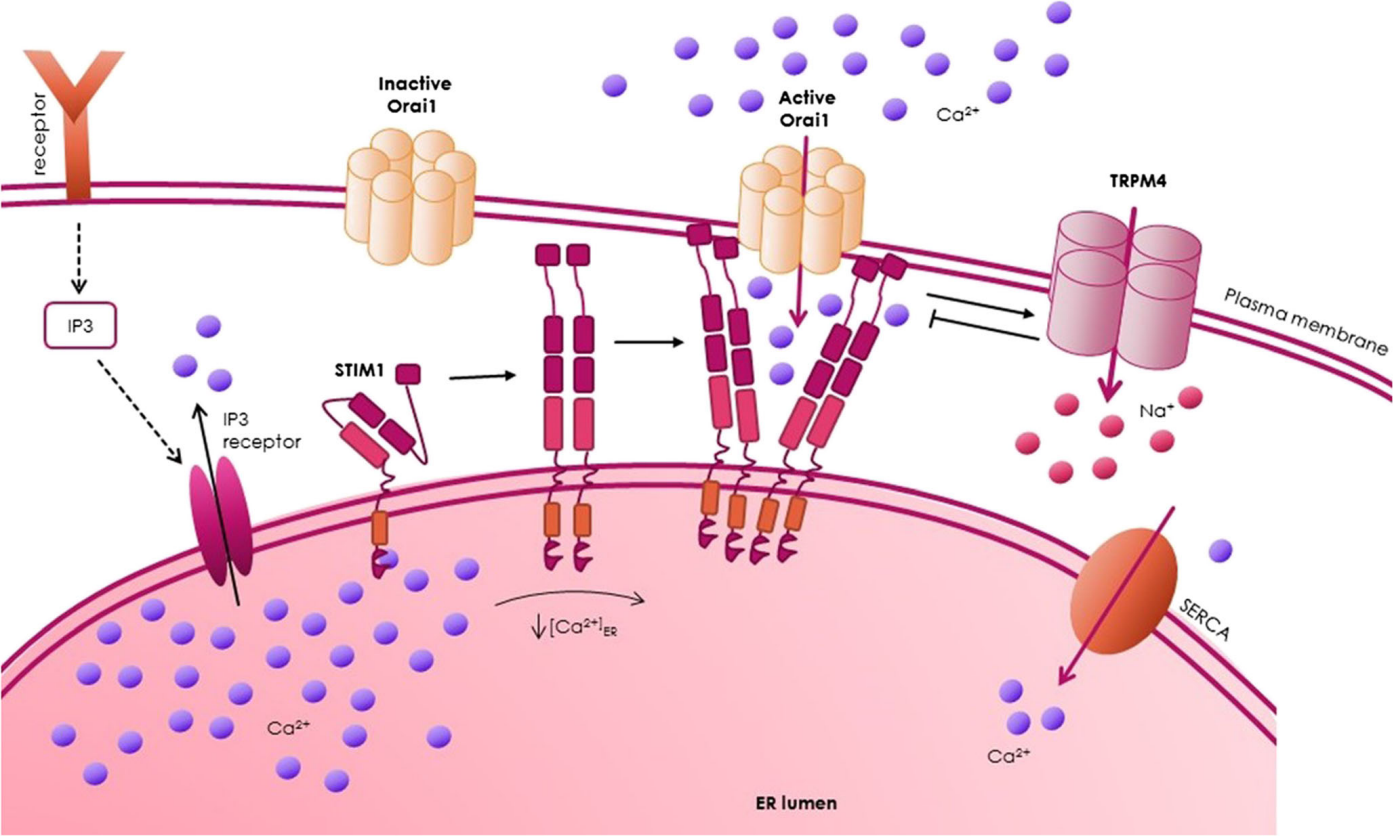
Activation of SOCE and TRPM4 as feedback mechanism for SOCE. Upon receptor stimulation, IP_3_ is produced and binds to the IP_3_ receptor. Subsequently, Ca^2+^ is released from intracellular Ca^2+^ stores. The decrease in Ca^2+^ in the ER results in the dissociation of Ca^2+^ from STIM1 proteins, which cluster and recruit and activate Orai1 channels in the plasma membrane that mediate SOCE. The increase in intracellular Ca^2+^ activates TRPM4. The Na^+^ influx via TRPM4 is a negative feedback mechanism for SOCE (please see text for details)

In order to mobilize Ca^2+^ for signaling, many cellular pathways lead to the production of inositol *1*,*4*,*5-*trisphosphate (IP_3_) as a second messenger. IP_3_ binds to IP_3_ receptors in the ER membrane that release Ca^2+^ from the ER. Upon the drop in the Ca^2+^ concentration, Ca^2+^ dissociates from the EF motif of stromal interaction molecule 1 (STIM1). This leads to STIM1 clustering, to the recruitment of Orai1 channels, and subsequently to store-operated calcium entry (SOCE) [9–11] (Fig. 1). SOCE affects cellular functions such as gene expression and cell proliferation, apoptosis, and migration, and several studies report the dysregulation of SOCE in cancer [12–15]. Specifically, the dysregulation of distinct molecular components of SOCE, especially the STIM1 and Orai1 proteins and their homologues STIM2, Orai2, and Orai3, plays important roles in the pathophysiology of different types of cancer. For example, dysregulation of both STIM1 and Orai1 contribute to human glioblastoma invasion [16]. In colorectal cancer, decreased STIM2 protein levels contribute to decreased Ca^2+^ concentrations in intracellular Ca^2+^ stores, and elevated SOCE is associated with increased cell proliferation and invasion and with characteristics that are implicated in tumor cell survival [17]. In an acute myeloid leukemia cell line, Orai1-Orai2 complexes mediate Ca^2+^ influx, which is important for cell migration [18]. Furthermore, STIM2 and Orai1 are the predominant isoforms in melanoma. These proteins contribute to adaptive tanning, while their dysregulation leads to the proliferation and migration of melanoma cells [19–22]. In addition, Orai1 is upregulated in breast cancer upon stimulation of the membrane androgen receptor [23]. The role of Orai3 has been extensively investigated in different types of cancer including lung [24, 25], breast [26–28], and prostate cancer [29, 30].

Many proteins regulate SOCE, including α-SNAP, CRACR2A, golli, ORMDL3, SARAF, septins, STIMATE, and TMEM110 [31–42]. In addition to direct modulation of SOCE by its key players, other regulatory mechanisms can influence SOCE. These mechanisms include pH modulation [43–45] and post-translational modifications like STIM1 phosphorylation and Orai1 glycosylation [46–50]. Na^+^ influx via the transient receptor potential melastatin-4 channel (TRPM4) [51, 52] decreases the driving force for Ca^2+^, which also regulates SOCE.

This article summarizes what is known about the dysregulation of the key molecular players involved in SOCE (STIM1, STIM2, Orai1, Orai2, and Orai3) and the negative regulator TRPM4 in prostate cancer. In addition, we introduce prostate cancer research models that allow the selection of a subpopulation of metastasis-initiating cells (MICs) with high metastatic potential. Finally, we introduce novel mouse and zebrafish prostate cancer research models. Future investigation of SOCE in these models will shed light on SOCE as a possible therapeutic target in prostate cancer.

## Gene Expression Levels of SOCE Components in Prostate Cancer

Table 1 summarizes studies [66] that compared gene expression in normal prostate tissue versus prostate carcinoma tissue. For Orai1, 5 out of 7 studies [53–57] reported a slight elevation in *ORAI1* gene expression in prostate cancer tissue, only 2 out of 12 studies [53, 54] reported an elevation in *ORAI2* gene expression. Notably, no changes in *ORAI3* gene expression levels have been detected in prostate cancer [56, 58]. For STIM1, 2 of 16 studies [59, 67] showed slightly elevated levels of *STIM1* gene expression in prostate cancer tissue, while 2 out of 5 studies [53, 54] reported elevated or slightly elevated gene expression levels for *STIM2*. These results suggest that some SOCE components may be dysregulated in prostate cancer.

**Table 1:** Gene expression levels of SOCE components and of the negative feedback regulator TRPM4 according to the indicated studies. The fold change relative to healthy prostate tissue, *p* values, and color-coding information are from Oncomine.org. Italic values indicate findings of significantly elevated gene expression levels in prostate cancer tissue, while italic-bold values indicating greater increases in expression. Unformatted values indicate that there was no significant change in gene expression in prostate cancer

Study	[53]	[54]	[55]	[56]	[57]	[58]	[59]	[60]	[61]	[62]	[63]	[64]	[65]
Number of patients	***21***	***19***	***30***	***185***	***40***	***101***	***122***	***89***	***112***	***35***	***34***	***102***	***57***
Orai1	*1.311* *0.033*	*1.354* *0.069*	*1.345* *0.047*	*1.062* *0.016*	*1.1046* *0.002*	1.062 0.348	1.004 0.478	–	–	–	–	–	–
Orai2	***1.683*** ***< 0.0005***	***1.281*** ***0.007***	-1.397 0.669	1.012 0.202	1.116 0.036	1.173 0.090	***–***	1.460 0.033	1.050 0.053	-1.148 0.592	-1.202 0.607	-1.392 0.960	-1.392 0.960
Orai3	–	–	–	1.002 0.433	***–***	-1.243 0.999	–	–	–	–	–	–	–
TRPM4	***2.761*** ***0.0001***	***2.002*** ***< 0.0001***	*1.940* *0.02*	*1.180* *0.0006*	***3.937*** ***< 0.0001***	***3.059*** ***< 0.0001***	–	***4.542*** ***0.0002***	–	–	–	–	***2.753*** ***< 0.0001***

## Protein Expression Levels of Orai1 and Orai3

STIM1 and Orai1 expression levels are differentially regulated depending on the prostate cancer stage [68]. In early clinically localized cancer stages, STIM1 and Orai1 expression is increased, while in the later castration-resistant prostate cancer stages, their expression levels are decreased. These findings are consistent with the known role of Orai1 in cell migration [69]. Furthermore, low Orai1 expression may contribute to an apoptosis-resistant phenotype in prostate cancer cells [70].

The expression levels of STIM1 and Orai1 correlate with the expression level of the androgen receptor [68]. Notably, STIM1 expression is directly regulated by androgens, and thus androgens can directly decrease Ca^2+^ signaling via STIM1 [71]. Remarkably, in breast cancer, estrogen receptor-positive breast cancer cells are associated with elevated levels of Orai3, which is an estrogen receptor α-regulated Ca^2+^ channel [26, 28]. Interestingly, an estrogen receptor-α blocker reduces the viability of prostate cancer cells [72]. These studies imply that there may be a direct link between estrogen receptor-α antagonists, SOCE, and cell viability.

Dubois et al. reported that the *Orai3* gene expression level was increased in 15 prostate cancer tissue samples compared to normal prostate tissue samples [30]. In that study, siRNA-based knockdown of *Orai3* did not change SOCE. The study suggested that native Orai1 and Orai3 proteins form non-store-activated ion channels that are activated by arachidonic acid [73, 74].

A study by our group found a slightly decreased Orai3/Orai1 ratio in prostate cancer tissue compared to normal prostate tissue [29]. Submaximal activation with an endogenous stimulus of SOCE, dihydrotestosterone, decreased SOCE signals upon siRNA knockdown of *Orai3*. In addition, the Orai3/Orai1 ratio correlated with the pharmacological profile of SOCE channels. First, application of 2-APB, which blocks currents via Orai1 and enhances currents via Orai3 [75], resulted in stronger amplification of I_CRAC_ in primary human prostate epithelial cells (hPECs) from healthy tissue compared to prostate cancer cells [29]. Second, reactive oxygen species (ROS) blocked SOCE and I_CRAC_ to a greater extent in prostate cancer cells than in primary human prostate epithelial cells from healthy tissue [76]. Orai1 is sensitive to ROS [77, 78], and ROS production seems to be coupled to Orai1 [79, 80]. In contrast, Orai3 is insensitive to ROS due to the lack of the ROS sensor, cysteine-195, that is present in Orai1 [77]. Furthermore, STIM2 appears to contribute to the ROS profile of SOCE [81]. Taken together, these findings show that the Orai1/Orai3 ratio is higher in prostate cancer cells than in healthy tissue, which is consistent with the observation that Orai1 is elevated in early clinically localized cancer stage [68].

Studies of *Orai3* expression level have produced inconsistent results, which may be due to the heterogeneous nature of prostate cancer. While our study focused on prostate cancer with Gleason scores of 6–8 [29], Dubois et al. excluded tumor tissue fragments that showed a mix of normal and tumoral tissue, which may have resulted in the selection of tissue samples from later stage prostate cancers [30]. To address this issue, the heterogeneity of prostate cancer must be taken into account. Future prostate cancer therapies may be personalized, with medicine that differentiates patients based on their genetic backgrounds and prostate cancer markers. Notably, great progress has been made in the development of tailored therapeutic approaches in prostate cancer [82].

## TRPM4

TRPM4 is a negative regulator of SOCE (Fig. 1) that contributes to the migration of dendritic cells, mast cells, and vascular endothelial cells [52, 83–87]. TRPM4 expression is associated with immune disease [88] and several cardiac diseases [89–100], with proliferation of breast cancer cells [101], and with poor outcome in B cell lymphoma [102]. While the database Oncomine reports no differences or only slight differences in the gene expression levels of STIM1, STIM2, Orai1, Orai2, and Orai3, TRPM4 expression is reported to be elevated in 8 out of 9 studies that compared its expression in cancer tissue samples versus normal or benign prostate tissue (Table 1). In addition, TRPM4 is a cancer-driver gene in androgen-insensitive prostate cancer [103•], and TRPM4 protein expression is upregulated in human prostate cancer tissue [104•, 105•]. Patients with higher expression levels of TRPM4 in prostate cancer glands compared to matched benign glands have an increased risk of biochemical recurrence [104•]. We previously demonstrated that siRNA-based knockdown of TRPM4 increases SOCE (Fig. 1) and reduces cell migration in the prostate cancer cell lines DU145 and PC3 [105•, 106]. In addition, Sagredo et al. recently showed that TRPM4 knockdown significantly reduce the proliferation of PC3 cells [107]. Thus, TRPM4 represents an interesting putative target in prostate cancer therapy.

## Future Cancer Research Models

The STIM and the Orai proteins are putative targets for cancer therapy [108, 109], and TRPM4 was more recently identified as a potential target for prostate cancer therapy. SOCE, and particularly the expression levels and functions of STIM and Orai proteins in prostate cancer, is complex and remains incompletely understood. Below, we introduce selected sophisticated prostate cancer research models, including cancer stem cell and mouse and zebrafish models that may increase our understanding.

## Cancer Stem Cells as a Cellular System for Studying Human Prostate Cancers

The study of ion channels in selected cell subpopulations may be a key strategy for identifying the biological functions of these molecules in order to understand their roles in prostate cancer maintenance and progression. According to the so-called cancer stem cell (CSC) hypothesis, only a selected subpopulation of cells supports tumor initiation and metastasis. Moreover, current therapies may efficiently target more differentiated “bulk” tumor cells without affecting the tumor- and metastasis-initiating properties of CSCs or metastasis-initiating cells (MICs). Determining the molecular characteristics of selected CSCs and MICs may help researchers formulate strategies to block cancer progression and metastasis.

One potential strategy for identifying and isolating MICs involves measuring the aldehyde dehydrogenase (ALDH) enzyme activity with the ALDEFLUOR assay [110, 111••]. The assay defines two subpopulations of cells, namely ALDH^low^ and ALDH^high^ cells. The ALDH^high^ subpopulations that have been isolated from prostate cancer cell lines (e.g., C4-2B and PC-3M-Pro4) showed increased aggressiveness and invasion *in vitro*. Importantly, in PC-3M-Pro4Luc2 cells, the ALDH^high^ subpopulation has much higher bone metastasis-initiating potential than the ALDH^low^ subpopulation. ALDH1A1 expression is associated with advanced clinical stage and unfavorable prognosis in hormone-naïve prostate cancer [112]. Recent studies show that bulk unsorted prostate cancer cell lines with different metastatic abilities can be distinguished based on the presence of specific ion channels [113]. Selected MICs, which are characterized by elevated ALDH activity, play a crucial role in tumor initiation and metastasis in human prostate cancer [111••]. Thus, this model represents a promising alternative to bulk and heterogeneous cell lines for assessing the contribution of SOCE components to prostate cancer.

Notably, the use of cellular models that have different metastatic characteristics shows promise as a way to investigate the roles of STIMs, Orais, and TRPM4 in the metastatic process. Despite the predominant blastic response at the level of bone metastasis in prostate cancer patients, a fraction of cases shows lytic lesions. Therefore, the use of different cell lines with distinct metastatic phenotypes (i.e., lytic vs. blastic) might provide new insights into the roles of SOCE components in prostate cancer bone metastasis. Blastic cell lines, such as VCaP and C4-2B, or lytic prostate cancer cell lines, such as PC3 and DU145, are examples of some available models that can be used to better understand the contribution of ion channel signaling during the formation of lytic and blastic bone lesions.

## Mouse Models

The use of blastic and lytic cell lines in animal models of bone metastasis may elucidate the contributions of STIMs, Orais, and TRPM4 in preclinical settings [114]. Current mouse models of intra-osseous (IO) and intra-cardiac (IC) inoculation are excellent models for dissecting the metastatic cascade in the different steps of metastatic dissemination. IO inoculation of lytic or blastic cells could be used to study the roles of SOCE signaling in the growth of human prostate cancer cells in bone. Similarly, IC injection represents a state-of-the-art model for measuring the contribution of SOCE signaling in the metastatic cascade, i.e., in dissemination, survival in the circulation, extravasation, homing to the distant site, and establishment of a metastatic lesion. Furthermore, it was recently shown that these models represent a good source of information for understanding the metastatic process in terms of events in both the tumor and the host [115]. This approach allows the tumoral and stromal components to be studied separately in order to identify the contributions of ion channels to the maintenance of the supportive stroma at the metastatic site.

## The Zebrafish Xenograft Model for the Study of Human Prostate Cancers

The zebrafish has long been used as a model for cancer research, first for studying chemically induced cancers [116, 117] and then, at the turn of the century, as a genetic cancer model [118]. However, it was only in the last decade that it made its debut as an animal system for the study of the xenotransplantation of mammalian cancer cells. Xenotransplantation can be defined as the process through which organs or tissues of one species are grafted or transplanted into another species. The method has long been used in cancer research, the first time by Green in 1938, who successfully transplanted adenocarcinomas from rabbit into a guinea pig eye [119]. Currently, this technique is used in mouse models to study the various phases of cancer development and progression. Haldi et al. were the first to describe xenografts of mammalian tumors into zebrafish embryos as a way to study tumor development [120]. This work was followed by that of Nicoli et al., who used xenografts of mammalian cancer cells in zebrafish embryos to analyze tumor-induced neoangiogenesis [121].

Following these initial studies of cancer xenografts in zebrafish embryos, many others used this model to study different aspects of tumor formation and cancer development. The zebrafish model allowed the investigation of the behavior of primary human tumors, such as pancreatic, colon, and stomach tumors [122], primary leukemia [123], and prostate cancer [124••], as well as their response to genetic [125] and chemical therapies [126]. In the case of primary human tumors, the zebrafish model could prove very useful, especially for the examination of the metastatic nature of a primary human tumor, as well as a tool for toxicological testing of potential anti-cancer drugs [127–129]. For example, in the study published by Bansal and colleagues [124••], they use the zebrafish xenograft transplantation model to access how frequently prostate tumor-initiating cells are found in different prostate cancer cell lines as well as in primary tumors.

The zebrafish model has unique advantages compared to, for example, the mouse model, especially when zebrafish embryos are used. In particular, a single female can produce over 100 eggs in 1 day, providing a large number of individuals for experimental and control groups from a single male/female couple. Within 25 h, the larvae are still less than 2 mm in size but already have an established vascular plexus with a beating heart, and all organ primordia are set. Importantly, the larvae can be kept transparent until later developmental stages. This transparency can be either genetically selected [130] or chemically induced [131] using quantum dots [124••], which has proved to be very useful for long-term *in vivo* imaging. Xenotransplanted cancer cells can then be followed over time either by fluorescent labeling with vital dyes that are stable throughout a few cell divisions [132] or by creating transgenic cancer cell lines that express a fluorescent protein associated with a gene of interest. The images are easy to acquire using a simple fluorescent stereomicroscope. High-resolution images can also be obtained using more advanced microscopy techniques, such as confocal, spinning-disk, two-photon, or light-sheet microscopy [133]. These more advanced methods not only allow researchers to track cells over time, but also enable 3D reconstructions, providing researchers with a way to analyze metastasis formation *in vivo* in four dimensions (time plus the *x*, *y*, and *z* planes). Even in adulthood, albino zebrafish strains such as Casper [130] are nearly fully transparent. This presents a unique opportunity to follow *in vivo* tumor development and progression. Furthermore, since embryos do not develop an adaptive immune system until 14 days post-fertilization [134], there is no need to suppress the immune system to facilitate the acceptance of foreign tissue when implanting xenografts. This provides researchers with an ideal system for studying tumor growth, invasion, and metastasis and how the microenvironment can affect these processes in a small organism [135]. This system allows rapid testing of drugs and genetic factors that might affect these processes [136] in order to identify ways to eradicate tumors [137, 138].

However, this model also has its limitations, and these must be taken into account when translating the acquired knowledge into other organisms, especially humans. Despite the similarity between the human and zebrafish genomes, zebrafish do not have many of the genes that are associated with cancer in humans [139]. This poses challenges in determining both the function of such genes and the molecular mechanisms that they might affect. Another major limitation is the difference in homeostatic temperatures between fish cell lines (usually grown at 28 °C) and mammalian cancer cell lines (37 °C). As a way to bridge this gap in physiological growth conditions, after xenograft implantation, zebrafish embryos can be grown at higher temperatures (32–35 °C). This allows the mammalian cells to grow and develop in an environment that is more similar to their natural state and has not been found to affect normal zebrafish development [120]. The benefits and limitations of the zebrafish xenograph model have been extensively analyzed in a recent review by Drabsch et al. [140].

**Fig. 2:**
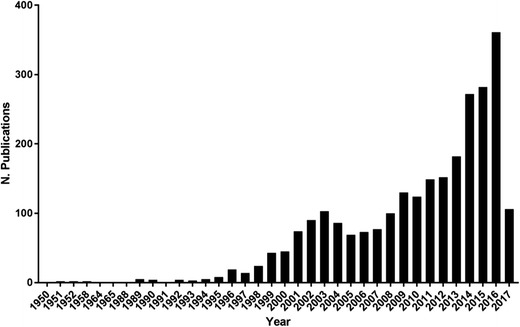
Evolution of cancer publications using the zebrafish model. The data were obtained from a PubMed search for “cancer + zebrafish”

In conclusion, although the zebrafish embryo xenograft model of cancer is not yet as well established as mouse models, it provides scientists with a rapid, cost-effective way to investigate tumor formation, microenvironment interactions, and new drug therapies, and it is an attractive option for testing personalized medicine. These benefits are reflected in the increasing number of cancer research publications that use the zebrafish as a model system (Fig. 2). Future studies of prostate cancer cell subpopulations using the zebrafish model are likely to give us valuable insights into prostate tumor development and metastatic behavior and will add to our understanding of the molecular mechanisms of SOCE.

## Conclusion

In the last decade, it has become increasingly clear that while SOCE signals impact the fates of prostate cancer cells, the underlying expression patterns and molecular mechanisms are complex and are not yet fully understood. Only a fraction of primary tumor cells are capable of recapitulating the neoplastic and malignant phenotype at a distant site, thus generating metastases. Therefore, selecting this subpopulation of cells from patient specimens and establishing cell lines that recapitulate the metastatic phenotype are promising as novel approaches to elucidate SOCE signaling in preclinical *in vitro* models. Cancer research models like the zebrafish model will add important insights in the *in vivo* setting. Additional studies are needed to investigate the dysregulation of ion channel signaling in more differentiated pathophysiological conditions using these novel cancer model systems.
