# Correction to: Store-Operated Ca^2+^ Entry as a Prostate Cancer Biomarker — a Riddle with Perspectives

**DOI:** 10.1007/s40610-018-0097-7

**Published:** 2018-06-20

**Authors:** Sven Kappel, Ines Joao Marques, Eugenio Zoni, Paulina Stokłosa, Christine Peinelt, Nadia Mercader, Marianna Kruithof-de Julio, Anna Borgström

**Affiliations:** 1 Institute of Biochemistry and Molecular Medicine, NCCR TransCure, University of Bern, Bern, Switzerland; 2 Institute of Anatomy, University of Bern, Bern, Switzerland; 3 Urology Research Laboratory, Department of Urology and Department of Clinical Research, University of Bern, Bern, Switzerland; 4 Department of Urology, Leiden University Medical Centre, Leiden, The Netherlands

**Correction to: Current Molecular Biology Reports (2017) 3:208–217**

10.1007/s40610-017-0072-8

The article "Store-Operated Ca^2+^ Entry as a Prostate Cancer Biomarker — a Riddle with Perspectives", written by Sven Kappel, Ines Joao Marques, Eugenio Zoni, Paulina Stokłosa, Christine Peinelt, Nadia Mercader, Marianna Kruithof-de Julio, and Anna Borgström, was originally published electronically on the publisher’s internet portal (currently SpringerLink) on October 28, 2017 without open access.

With the author(s)’ decision to opt for Open Choice the copyright of the article changed on June 2018 to © The Author(s) 2018 and the article is forthwith distributed under the terms of the Creative Commons Attribution 4.0 International License (http://creativecommons.org/licenses/by/4.0/), which permits use, duplication, adaptation, distribution and reproduction in any medium or format, as long as you give appropriate credit to the original author(s) and the source, provide a link to the Creative Commons license and indicate if changes were made.

The original article has been corrected.

